# Michigan State Board of Health

**Published:** 1879-03

**Authors:** 


### ARTICLE XII.

### MICHIGAN STATE BOARD OF HEALTH.

The regular quarterly meeting of the State Board of Health was held in Lansing, on Tuesday, Jan. 14, this being its first meeting in its rooms in the new capitol building. The following members were present: R. C. Kedzie, M.D., President; H. O. Hitchcock, M.D., Hon. LeRoy Parker, Rev. D. C. Jacokes, and Henry B. Baker, Secretary.

### ADULTERATION OF SUGARS, SYRUPS AND HONEY.

As committee on foods, drinks and water supply, Dr. Kedzie made a verbal report on table sweets, showing the methods of adulteration now practiced. One of these is by the use of glucose, which is an inferior article of sugar, formed by the action of sulphuric acid on starch. In sugars thus adulterated, there is usually found sulphuric acid and copperas. Another method lately practiced has been for the lessening of duties, and consists in coloring sugar, so as to make it appear of lower grade. The danger comes from the poisonous chemicals used in bleaching. Dr. Kedzie also mentioned the fact that where bees are fed on glucose, this substance will be deposited in the cells without change. In connection with the adulteration of sugar, the doctor also said that one bushel of corn would make about 40 pounds of grape-sugar or glucose. Where the sugar is of a blue tinge, it is an evidence that blueing has been added to the sugar to relieve the yellow appearance due to the adulteration. The experiments and reports heretofore made by Dr. Kedzie had been made the basis of a memorial to congress, asking legislation upon the subject.

### VENTILATION OF BUILDINGS ALREADY CONSTRUCTED.

The committee on the subject of ventilation and heating of buildings, Rev. Dr. Jacokes, read a paper on the heating and ventilation of buildings already constructed. He first showed the importance of good ventilation, and then pointed out many ways in which buildings already constructed, faulty in this particular, could be improved. One method was the leading of fresh air from outdoors to a jacket enclosing a space around a stove, and withdrawing the foul air from the floor level by means of pipes which lead from near the floor to the chimney above. He showed many diagrams illustrating the methods of improving the ventilation in buildings already constructed. He gave one illustration of a church which had been insufficiently warmed by three stoves, but which was afterwards thoroughly warmed and ventilated by one of these stoves, properly jacketed, and the cold and foul air withdrawn from the floor level. The ventilation of two churches by a similar method cost but $10, and ventilating apparatus for dwellings cost from $1.25 to $10.

### ILLUMINATING OILS.

As committee on special sources of danger to life and health, Dr. Kedzie made a verbal report on illuminating oils. In this report he brought before the Board a sample of "mineral seal" oil, a new brand, which stands a flash test of 260 degrees, by the Michigan method, and will also stand the Michigan chill test. He exhibited a lamp filled with this oil, which gave a brilliant light equal to twenty-six railroad candles. This oil is manufactured for the Standard Oil Co., in Cleveland. It sells for forty cents a gallon. It is made by freezing the paraffine out of heavy paraffine oil. He recommended the use of this oil on railroad cars under very stringent provisions. He showed the safety of the oil by heating it to 254 degrees and plunging lighted pine sticks into it, when they were immediately extinguished.

He also brought up for discussion the question as to what action the Board shall take with reference to illuminating oil for ordinary purposes. As showing the ways in which certain parties seek to evade our laws, he exhibited a specimen of oil and the dealers' circular in connection with it, claiming a new and safe illuminator called "potalene." It was on exhibition at the State Fair, pleading for a premium. He put fifteen drops in a heavy glass bottle and touched a lighted match to the mouth of the bottle, when the flames and smoke shot several feet high. He said this oil was simply naphtha in which potatoes had been soaked.

It was voted that the interests of life in this State will be subserved by maintaining the present tests for illuminating oils. Dr. Kedzie was requested to make a thorough investigation of the whole subject, and to act for the Board in endeavoring to maintain the present tests.

### REGULATION OF MEDICAL PRACTICE.

LeRoy Parker, committee on legislation in the interests of public health, read a report on the subject of the Illinois law regulating medical practice, and on the proposal to regulate the practice in Michigan. The Illinois law compels an examination by a State Board, and the effect is to drive quack doctors out of the

State, and some have come to Michigan. He recommended the enactment of a law by the Michigan Legislature, requiring practitioners to undergo examination.

Dr. Hitchcock presented the form of a memorial to the Legislature on the same subject, expressing the opinion that great injury is being done to the health of many persons in this State, and that many deaths occur because of treatment by ignorant and unscrupulous pretenders, bearing the name of doctor, with perhaps the title of "M. D." He recommended an examination of practitioners in anatomy, physiology and pathology of the human body, and in chemistry and botany.

The memorial was adopted.

### SANITARY TOPOGRAPHY.

LeRoy Parker made a verbal report on a memorial to the legislature on the subject of topographical survey of the whole State for sanitary purposes. Dr. Baker was added to the committee and it was recommended that the survey should not only be topographical, but should embrace all sanitary subjects, especially those relating to water, as, for instance, drainage, pollution of streams, water-supply, etc.

### SANITARY CONVENTION.

Dr. Baker, who was a committee to prepare for a sanitary convention at Coldwater, reported that owing to the death of Dr. J. H. Beech of that city, the local support to the movement was withdrawn, and it was impossible to hold the convention. Much work has been done in preparing for this meeting, and he had endeavored to secure such a meeting at some other place. A communication was received from Dr. J. H. Kellogg, of Battle Creek, inviting the Board to hold a meeting at that place, pledging the support of the citizens of that city to make the meeting a success. The subject was postponed to the April meeting.

A committee was appointed to draft resolutions relative to the death of Dr. Beech, and its chairman, Dr. Hitchcock, presented the following preamble and resolution which was adopted by the Board.

"WHEREAS, We have hoard with deep regret of the death of Dr. J. H. Beech, of Coldwater; therefore,

"*Resolved*, that in the death of Dr. Beech this Board has lost one of its most interested and efficient correspondents, and the people of the State have lost one of their most intelligent, earnest and practical sanitarians."

### DIPHTHERIA STATISTICS.

The Secretary presented reports from Dr. E. N. Palmer, of Brooklyn, Jackson County, relative to the outbreak of diphtheria in that section. During a period of five months, there were sixty-seven cases and eleven deaths. He gave several instances where diphtheria had been communicated by persons convalescent from that disease; also, by persons who did not have the disease at all, but were in attendance on patients.

The document on the prevention and restriction of diphtheria, issued by this Board, has been in great demand, not only in Michigan, but throughout other States and Territories.

### DRAINS AND SEWERS.

Dr. Baker presented a communication from A. C. Sekell, City Surveyor of Grand Rapids, transmitting rules and regulations of the Board of Public Works, in that city, with reference to house-drains and sewers, and moved for the preparation of a circular asking the facts concerning the regulation of drains and sewers in the cities of Michigan, and also the views of sanitary experts and engineers, with a view to making some general recommendations for the benefit of the many small cities and villages now just beginning to consider the subjects of drains and sewers.

By request of members of the Board of Control of the State public school, Dr. Baker presented the subject of the temporary maintenance of diseased and crippled children at the State Hospital in Ann Arbor, as a means of preventing sickness and pauperism in after life.

After the auditing of bills incurred during the past quarter, and the transaction of other necessary business, the Board adjourned to the annual meeting, April 8, 1879.

